# Effects of supplemental hybrid bacterial 6-phytase in low-energy, inorganic phosphorus-free hen diets on laying performance, egg quality, and bone strength

**DOI:** 10.1371/journal.pone.0322135

**Published:** 2025-04-30

**Authors:** Pinar Sacakli, Muhammad Shazaib Ramay, Ahmet Ceylan, Özge Özgenç Çınar, Fatma Kübra Erbay Elibol, Josoa André Harijaona, Yauheni Shastak, Peter Ader, Dieter Feuerstein, Ali Calik

**Affiliations:** 1 Department of Animal Nutrition and Nutritional Diseases, Faculty of Veterinary Medicine, Ankara University, Ankara, Türkiye; 2 Department of Histology Embryology, Faculty of Veterinary Medicine, Ankara University, Ankara, Türkiye; 3 Department of Micro and Nanotechnology, TOBB University of Economics and Technology, Ankara, Türkiye,; 4 BASF SE, Ludwigshafen, Germany.; University of Agriculture Faisalabad, PAKISTAN

## Abstract

A 22-week trial was conducted to assess the effects of replacing inorganic phosphorus (P) with two levels of a hybrid bacterial 6-phytase in low-energy diets for laying hens, from 23 to 44 weeks of age. The study focused on hen performance, egg quality and bone health of laying hens. For this purpose, Lohmann Brown Classic hens (n = 432) were randomly allocated to four dietary groups, each comprising nine replicates of 12 birds. The groups included: (1) positive control (PC), a standard diet containing 3.7% calcium, 0.38% non-phytate phosphorus (nPP) and 2730 kcal/kg metabolizable energy (ME), (2) negative control (NC), a diet similar in nutritional specifications to the PC but with reduced nPP (0.12%) and ME (2630 kcal/kg), (3) NC300 and (4) NC600, where NC diets were supplemented with 300 and 600 phytase unit (FTU) per kg feed, respectively. All diets were provided as mash and formulated using corn, soybean meal and sunflower meal as the main ingredients. The NC diet significantly impaired hen performance compared to the PC diet (p < 0.05). Specifically, the NC diet led to deterioration in egg production (p < 0.001), egg weight (p = 0.001), egg mass (p < 0.001), feed intake (p < 0.001), feed conversion ratio (p = 0.002), body weight (p < 0.001), and livability (p = 0.036). Additionally, the NC diet increased the incidence of cracked (p < 0.001) and shell-less eggs (p < 0.001) and lowered eggshell breaking strength (p = 0.005). Bone health was also adversely affected by the NC diet, as indicated by reduced tibia ash content (p < 0.001), stiffness (p = 0.005), and maximum load-bearing capacity (p = 0.040). Moreover, with NC diet, there was a decrease in osteoprotegerin (OPG) expression (p < 0.001) and an increase in receptor activator of nuclear factor kappa-B ligand (RANKL) expression (p < 0.001) in tibia, resulting in a greater RANKL/OPG ratio (p < 0.001). Supplementing the NC diet with bacterial 6-phytase at both levels (300 and 600 FTU/kg) effectively mitigated all adverse effects of P and ME deficiency on the aforementioned parameters, bringing them to levels comparable to those of the PC. Notably, the 600 FTU/kg supplementation provided slightly better results in terms of egg weight and eggshell breaking strength than the 300 FTU/kg level. Overall, this study suggests that supplementing the hybrid bacterial 6-phytase (300–600 FTU/kg) to P-deficient (0.12% nPP) and low energy (−100 kcal/kg) diets can fully replace inorganic P without compromising laying performance, egg quality, or bone health. Further research is recommended to determine the optimal levels of hybrid bacterial 6-phytase in P-deficient diets for laying hens throughout laying cycle of the birds with other nutrient matrices (energy, amino acids, calcium) to optimize layer feed formulations.

## Introduction

Phosphorus (**P**) is an essential macro-mineral required for optimal poultry production. It plays a critical role in skeletal health and egg formation, while also supporting key biochemical functions such as energy metabolism, amino acid metabolism, protein synthesis, and the regulation of osmotic and acid-base balance in poultry [1]. However, two-thirds of the total phosphate in plant-based diets for monogastric animals, including poultry, is stored as phytate-P and excreted undigested [2]. Phytate-P is poorly absorbed in the gastrointestinal tract of monogastric animals and it can negatively affect the digestibility of other nutrients and overall performance due to its anti-nutritional properties [3–5].

Over the past few decades, exogenous phytases have been widely used in poultry nutrition to counteract the anti-nutritional effects of phytate. Phytase enzyme improves the utilization of P and calcium [6,7], enhances nutrient digestibility, and reduces environmental pollution by lowering P excretion in poultry manure [8]. Phytase increases the amount of hydrolyzed phytate P available to birds by breaking down the phosphodiester bonds in phytates. Thus, dietary supplementation with exogenous phytases can reduce the requirement for non-phytate P (**nPP**), thereby decreasing reliance on costly and non-renewable inorganic P reserves [3,9,10]. Additionally, phytate forms indigestible complexes with other minerals, starch, proteins, thereby negatively affecting nutrient digestion and the energy utilization of diets [11]. Previous studies have reported that, in addition to increasing P availability, phytase can enhance nutrients availability and improve energy digestibility in laying hens [12,13]. As a result, phytase supplementation can help reduce the dietary metabolizable energy **(ME**) without negatively impacting hen performance. Research suggests that the benefits of phytase are most pronounced when incorporated into low-energy poultry diets [14,15]. Studies have shown variable results, with reductions in dietary ME of laying hens ranging from 50–75 kcal/kg while maintaining performance [8,15–17]. However, these outcomes depend on several factors, including P-availability, phytase dosage and the age of the birds.

For laying hens, the recommended nPP requirement without phytase supplementation was previously estimated at 0.25% [18]. However, numerous studies have demonstrated that nPP levels below this threshold can still support laying hen health and productivity [19,20], and the addition of phytase could allow for further reductions in these levels [21]. Optimal dietary nPP levels for laying hens have been identified as 0.18%, 0.15%, and 0.14% when the diet contained phytase at 150, 300, and 400 FTU/kg, respectively [22]. Despite the evident benefits of exogenous phytase, several challenges related to its effective utilization persist. Previous works, with comparable dietary nPP levels, have shown contradictory results. Some studies suggest that 300 FTU/kg of phytase is sufficient to reduce nPP to 0.12–0.15% and eliminate inorganic P from hen diets without compromising performance [19,22], while others indicate that 300 FTU/kg is insufficient, particularly for maintaining egg production [17]. Further, Lei et al. [16] found minimal improvement in the feed conversion ratio (**FCR**) with phytase supplementation, despite enhanced nutrient availability and laying performance, whereas Pirzado et al. [15] reported that phytase significantly improved FCR, even surpassing the standard diet. Similarly, contradictory results have been observed for eggshell [15,17,23,24] and bone [23,25,26] quality characteristics when phytase is supplemented in laying hen diets. These variations in results across studies may be attributed to differences in phytase efficacy, enzyme quality, diet composition, bird type, age and physiological condition [27,28]. Technological advancements in phytase development have paved the way for the creation of next generation products with enhanced stability and performance [29,30] and a wide range of commercial exogenous phytases, each derived from distinct production strains, is now available. This study aimed to assess the potential of a hybrid novel consensus bacterial 6-phytase variant to replace dietary inorganic P in low-energy laying hen diets (from 23 to 44 weeks of age) and to evaluate its impact on hen performance, egg quality, and bone parameters.

## Materials and methods

### Phytase enzyme

The bacterial derived 6-phytase used in this study, (Natuphos E® 10000, BASF SE, Germany), is a hybrid enzyme assembled from three bacterial species *Hafnia*, *Yersinia*, and *Buttiauxella-* and expressed in *Aspergillus niger*.

### Birds and management

This project was approved and conducted under the guidelines of the Animal Care and Use Committee of Ankara University (2021-18-166). A total of 432 Lohmann Brown Classic hens, aged 23 wk, were randomly allocated to four experimental groups, using nine replicates each and 12 hens/replicate. The experiment included two control groups: a positive control (**PC**) with standard levels of dietary nPP and ME, and a negative control (**NC**) with suboptimal nPP and ME to assess the effects of nutrient deficiency. Additionally, two NC groups were supplemented with phytase to evaluate whether phytase could restore nutritional adequacy to the level of a standard diet. The hens were weighed before being placed in cages, and the trial spanned for 22 weeks, starting at 23-wk and concluding at 44-wk of age. The experiment was divided into five periods, i.e., four periods of 4 wk each and fifth period of 6 wk (Period 1, 23–26 wk; Period 2, 27–30 wk; Period 3, 31–34 wk; Period 4, 35–38 wk; Period 5, 39–44 wk).

The hens were raised in stainless steel cages located in an environmentally controlled facility. Hens in each replicate had access to nipple drinkers, an external feed trough and an egg tray. Feed and water were provided *ad libitum*. Standard management practices were implemented in accordance with the Lohmann Brown-Classic management guide. The facility was artificially ventilated, and a lighting schedule of 16L:8D was maintained throughout the experiment.

### Experimental diets

In the experiment, the first treatment group, the PC, was fed corn, soybean meal, and sunflower meal based basal diet containing 0.38% nPP and 2730 kcal/kg ME. The PC group received inorganic P from di-calcium phosphate, with no added phytase, and was formulated to meet or exceed Lohmann Brown Classic nutrients recommendations with daily feed consumption of 110 g/hen/day. The second treatment group, NC, shared similar nutritional specifications with the PC diet but had reduced dietary ME concentration (2630 vs. 2730 kcal/kg) and nPP level (0.12 vs 0.38%). The NC diet was formulated without any inorganic P supplement and did not include phytase. The third and fourth treatment groups received the NC diet supplemented with bacterial 6-phytase at two levels: 300 (**NC300)** and 600 (**NC600)** FTU/kg feed, respectively. Phytase was prepared as a premix using ground corn and included in the diets at 50 g/kg. The inclusion levels of phytase in each diet were adjusted based on the analyzed activity of the phytase product. The composition of PC and NC diets is provided in Table 1. All experimental diets were offered as mash and analyzed for dry matter and crude protein (Kjeldahl procedure) [31]. Additionally, the diets were analyzed calorimetrically for their calcium [31] and phosphorus composition [32].

**Table 1:** Ingredients and composition of basal diet (as-fed basis).

Ingredient, %	PC	NC
Corn, CP 7.2%	58.50	58.0
Soybean meal, CP 47%	21.0	15.0
Sunflower meal, CP 35%	7.50	15.40
Soybean oil	1.85	1.00
Limestone	8.53	9.63
Di-calcium phosphate	1.74	–
DL-Methionine (98%)	0.19	0.18
L-Lysine HCl	0.04	0.14
Sodium Bicarbonate	0.20	0.20
Sodium chloride	0.25	0.25
Vitamin Premix^1^	0.10	0.10
Mineral Premix^2^	0.10	0.10
**Calculated Composition**		
Dry Matter, %	89.55	89.58
Crude Protein, %	16.85	16.85
AME_n_, kcal/kg	2730	2630
Lysine, %	0.86	0.86
Dig. Lysine, %	0.74	0.75
Methionine + cysteine, %	0.76	0.77
Dig. Methionine + cysteine, %	0.68	0.68
Threonine, %	0.64	0.63
Dig. Threonine, %	0.54	0.53
Calcium, %	3.73	3.74
Total Phosphorus, %	0.67	0.41
Non-Phytate Phosphorus, %	0.38	0.12
**Analyzed Composition**		
Crude Protein, %	16.78	16.82
Calcium, %	3.73	3.79
Total Phosphorus, %	0.69	0.42

PC, positive control; NC, negative control; CP, crude protein; AME_n_, available metabolizable energy corrected for nitrogen

^1^Provided per kilogram of complete diet: vitamin A, 15,000 IU; vitamin D3, 5,000 IU; vitamin E, 100 mg; vitamin K3, 3 mg; thiamin, 5 mg; riboflavin, 8 mg; pyridoxine, 5 mg; pantothenic acid, 16 mg; niacin, 60 mg; folic acid, 2 mg; biotin, 200 µg; vitamin B12, 20 µg.

^2^Provided per kilogram of complete diet: Cu, 16 mg; I, 1.5 mg, Co, 500 µg; Se, 350 µg; Fe, 60 mg; Zn, 100 mg; Mn, 120 mg; Mo, 1 mg.

### Performance

Individual body weights were recorded at the beginning (wk 23) and end (wk 44) of the experiment. Mortality and the total number of eggs were recorded daily, including dirty, cracked and shell-less eggs. For each experimental period, leftover feed and eggs were weighed and the hen-day egg production (**HDEP**, %), hen-house egg production (**HHEP**, %), feed intake (**FI**, g/bird/day), egg mass (HDEP × egg weight, g), and FCR (g feed/ g egg mass) were calculated and corrected for mortality.

### Egg quality

At the end of each experimental period, two eggs per replicate were randomly collected (18 eggs/treatment) and weighed. Egg shape index [(egg width/ egg length) × 100] was measured using a specialized instrument (B.V. Apparatenfabreik Van Doorn, No: 75 135/2, De Bilt, Holland). Eggshell breaking strength was determined using the egg-breaking tester of static compression device (Dr.-Ing. Georg Wazau Mess-und Prüfsysteme GmbH, Berlin, Germany). Later, the contents of each egg were broken onto a table. The height of the albumen and yolk were measured using a tripod micrometer (Mitutoya, No. 2050-08, 0.01–20 mm; Kawasaki, Japan). The length and width of the albumen, as well as yolk diameter were measured using a digital caliper. Using these measurements, indices of egg freshness were calculated, including yolk index [(yolk height/ yolk diameter) × 100], albumen index [(albumen height/ average of albumen length and albumen width) × 100] and Haugh units [100 log (albumin height + 7.57–1.7 W^0.37^), where W is egg weight]. Yolk color was assessed using the Roche yolk fan. Eggshell thickness was measured at three different points (top, middle and bottom) using a micrometer (Mitutoya, No. 1044N, 0.01–5 mm; Kawasaki, Japan). The average of these three measurements was then calculated and recorded as the eggshell thickness. Egg quality parameters detailed above were determined as described elsewhere [33].

### Tibia sampling

At the end of the experiment (44 wk), two hens from each replicate (18 hens/treatment) were randomly selected and euthanized by cervical dislocation. Right and left tibia samples were collected, cleaned of any adherent soft tissues and prepared for subsequent analyses. The right tibia samples were divided into two groups of nine each for the evaluation of bone breaking strength and immunohistochemical analysis.

### Tibia ash content

All left tibia samples (18 hens/treatment) were defatted by ether extraction (Buchi E816 SOX, Flawil, Switzerland) for 12 h, and later dried in hot air oven (105 °C) for 24 h [34]. Dry bone weights were recorded, and bone samples were ashed using a muffle furnace at 600 °C for 8 h [35].

### Bone breaking strength

Right tibia samples (9 hens/treatment) were subjected to breaking strength measurement based on a three-point bending test on Instron 5944 testing frame (Instron, Norwood, MA, USA). Load was applied from the mid-shaft of tibias. Span length was 60 mm and loading rate was 5 mm/min. Tests were performed until failure occurred. During tests, load and displacement data was collected. Load-Displacement curves were created by using load and displacement values. Stiffness values were calculated by using slope of Load-Displacement curve. Bone breaking strength refers to the maximum load that a sample can withstand before failing [36].

### Immunohistochemical analysis of osteoprotegerin (OPG) and receptor activator of nuclear factor kappa-B ligand (RANKL) in tibia

Right tibia samples (9 hens/treatment) were used for immunohistochemical analysis. Transverse sections from the medial part of tibia were collected and immersed in 10% neutral-buffered formalin for 72 hours, following standard fixation protocols [37]. Subsequently, the bone specimens were trimmed to appropriate sizes to facilitate effective penetration of decalcifying agents. The five-week decalcification was performed at room temperature using 10% formic acid. To ensure consistent and effective decalcification, the decalcifying solution was refreshed daily, preventing saturation and maintaining its efficacy. Additionally, continuous mechanical agitation was applied throughout the procedure to promote uniform exposure of the tissue to the decalcifying agent, thereby enhancing the diffusion process and preventing uneven decalcification [38]. Following decalcification, bone samples were thoroughly rinsed with running water to remove residual decalcifying agent. Decalcified bone specimens then underwent standard histological processing techniques, including dehydration with graded alcohol, clearing with xylene, and embedding in paraffin. Sections (7 μm) were cut from the paraffin-embedded bone blocks using a microtome (Leica RM2235) and sections were transferred onto poly-L-lysine-coated slides. After incubation at 37 ºC overnight in an oven, the sections underwent deparaffinization and rehydration processes and were placed in distilled water. Following this stage, the sections were treated with 3% H_2_O_2_ (prepared with methanol) to eliminate endogenous peroxidase activity and were left for 20 min and then rinsed in distilled water for 5 min. For antigen retrieval, the sections were transferred to plastic microwave vials containing 0.01M citrate buffer (pH 6.0). The vials were heated in a microwave oven at 600W for four consecutive 5-minute cycles, with the buffer level checked and replenished with distilled water as needed. Afterward, they cooled at room temperature for 20 min. Then sections were washed with phosphate buffer solution (PBS) for 5 min and outlined with PAP-PEN.

Sections were incubated with 10% normal mouse serum for 10 min at room temperature to block non-specific binding, followed by overnight incubation with primary antibodies (anti-RANKL, Bioss-bs-0747R, 1:300, and anti-OPG, Bioss-bs20624R, 1:300) at +4 ºC. Negative controls were included by omitting the primary antibody. Then, the sections were treated with biotinylated secondary antibody for 30 min, followed by incubation with streptavidin-horseradish peroxidase (Histostain Plus, Zymed kit: 85–6743, USA) for 30 min at 37 ºC in a humid environment. The sections were washed with PBS for 10 minutes before each incubation. Afterwards, the sections were treated with diaminobenzidine chromogen substrate and washed with tap water. Finally, the sections were counterstained with Gill’s hematoxylin, dehydrated, and covered with Entellan. All findings were evaluated and imaged using a light microscope (Leica DM 2500, Leica Microsystems GmbH, Wetzlar, Germany) and a camera (Leica DFC 450, Leica Microsystems GmbH, Wetzlar, Germany). Immunolabeling for RANKL and OPG was analyzed in the cross sections of tibias under ×400 magnification. To assess potential differences in the pattern of immunolabeling between groups, a quantitative analysis was performed using ImageJ. Immunopositive cells were quantified in three randomly selected bone tissue sections encompassing the entire cross-section of the tibia, without regard to the intensity of staining. One blinded, trained examiner selected the sections for histometric analysis. Another masked, calibrated examiner conducted the histometric analysis. The values for each section were measured three times by the same examiner on different days to reduce variations in the data.

### Statistical analysis

Statistical analysis was conducted using SPSS software (V22.0; SPSS Inc., Chicago, IL, USA). One-way analysis of variance (ANOVA) was performed for variables including egg production, performance, egg and tibia quality characteristics. The results were presented as means and pooled standard error of mean. Tukey test was employed when there were significant differences among treatments. Arcsine transformation was applied to data expressed as percentages (except for mortality). Mortality rates were compared using a chi-square test. Statistical significance was set at p ≤ 0.05.

## Results

### Egg production, egg weight, and egg mass

The results of egg production (HHEP, HDEP), egg weight and egg mass are presented in Table 2. No significant differences in HHEP and HDEP were observed among treatment groups during the first two periods (23–26 wk and 27–30 wk) of the study. However, hens on the NC diets showed a decline in production performance compared to the PC group (p < 0.05) from 31^st^ week. Supplementing NC diets with phytase at 300 and 600 FTU/kg effectively mitigated decline in egg production. Both phytase doses rectified the adverse effects of reduced P and energy level on overall egg production (23–44 wk) (p < 0.001).

**Table 2:** The effects of phytase supplementation on HHEP, HDEP, egg weight and egg mass in hens on low phosphorus and low-energy diets^1^.

	Treatment Groups^2^				Statistics	
Item^3^	PC	NC	NC300	NC600	SEM	p-value
**HHEP, %**						
23-26 wk	91.22	93.70	91.88	92.44	0.94	0.686
27-30 wk	95.28	93.83	96.33	97.56	0.61	0.122
31-34 wk	95.77^ab^	90.56^b^	97.04^a^	96.94^a^	0.89	0.020
35-38 wk	94.59^a^	82.77^b^	95.46^a^	95.91^a^	1.26	<0.001
39-44 wk	89.39^a^	67.64^b^	92.44^a^	92.28^a^	2.09	<0.001
Overall	93.25^a^	85.70^b^	94.63^a^	95.03^a^	0.93	<0.001
**HDEP, %**						
23-26 wk	91.22	93.70	91.88	92.44	0.94	0.686
27-30 wk	95.57	95.50	96.97	97.74	0.44	0.079
31-34 wk	96.81^ab^	95.37^b^	97.96^a^	97.85^ab^	0.44	0.049
35-38 wk	96.96^a^	88.57^b^	97.35^a^	97.51^a^	0.85	<0.001
39-44 wk	93.91^a^	74.25^b^	95.38^a^	95.00^a^	1.78	<0.001
Overall	94.89^a^	89.48^b^	95.90^a^	96.11^a^	0.69	<0.001
**Egg Weight, g**						
23-26 wk	57.65^b^	58.57^a^	57.77^ab^	58.61^a^	0.16	0.050
27-30 wk	60.52	60.44	60.84	61.12	0.16	0.444
31-34 wk	62.04	61.89	61.55	62.20	0.22	0.779
35-38 wk	62.71	61.45	61.70	62.46	0.22	0.142
39-44 wk	62.56^a^	58.99^c^	60.92^b^	61.37^ab^	0.33	<0.001
Overall	61.25^a^	60.09^c^	60.47^bc^	60.80^ab^	0.12	0.001
**Egg Mass, g**						
23-26 wk	52.63	54.91	53.06	54.19	0.62	0.562
27-30 wk	57.84	57.72	58.99	59.74	0.32	0.076
31-34 wk	60.07	59.03	60.29	60.86	0.36	0.337
35-38 wk	60.81^a^	54.40^b^	60.06^a^	60.90^a^	0.60	<0.001
39-44 wk	58.74^a^	43.91^b^	58.09^a^	58.29^a^	1.24	<0.001
Overall	58.17^a^	53.83^b^	58.03^a^	58.46^a^	0.46	<0.001

a-c Means with no common letters in the same row differ significantly (p < 0.05).

^1^Data represent mean values of 9 replicates per treatment.

^2^PC, positive control (nutritionally adequate diet based on breeder nutrient recommendations); NC, negative control (the PC diet with reduced metabolizable energy 2630 vs. 2730 kcal/kg, and available phosphorus levels 0.12 vs 0.38%); NC300 and NC600, the NC diets supplemented with hybrid novel consensus bacterial 6-phytase (Natuphos E^®^ 10000) at 300 and 600 FTU/kg, respectively.

^3^HHEP, hen-house egg production; HDEP, hen-day egg production.

The results for egg weight demonstrated a significant increase during the first period (23–26 wk) in birds fed NC diets, both with and without phytase supplementation, compared to the PC group (p = 0.05). In the subsequent three periods (weeks 27–38), egg weights remained comparable across all treatments (p > 0.05). In the final period (39–44 wk), eggs from hens on the NC diets were lighter than those from the PC, NC300 and NC600 groups (p < 0.001). Overall (23–44 wk), the NC and NC300 groups produced lighter eggs compared to PC and NC600 groups (p = 0.001). During the last two periods (35–38 wk and 39–44 wk), as well as overall (23–44 wk), egg mass significantly decreased in hens fed NC diets compared to the PC group (p < 0.001). Supplementing the NC diets with phytase at both doses significantly improved egg mass, restoring it to levels comparable to the PC group (p < 0.001).

### Feed intake, feed conversion ratio, hen weights and mortality

The results for FI and FCR are presented in Table 3. During the initial period of the trial (23–26 wk), dietary treatments did not significantly affect FI (p > 0.05). However, between wk 27–30 and 31–34, hens consuming diets supplemented with phytase (NC300 and NC600) exhibited significantly higher FI (p < 0.001) compared to those fed PC and NC diets without phytase. Furthermore, from wk 35–38 and 39–44, a notable decrease in FI was observed in hens fed NC diets compared to the PC group (p < 0.001). The addition of phytase (NC300 and NC600) resulted in a comparable FI to hens fed PC diets (p < 0.001). Overall (23–44 wk), hens fed NC diets had lower FI compared to the PC group. The supplementation of phytase in the NC diets effectively mitigated this reduction (p < 0.001).

**Table 3:** The effects of phytase supplementation on feed intake and feed conversion ratio in hens on low phosphorus and low-energy diets^1^.

	Treatment Groups^2^				Statistics	
	PC	NC	NC300	NC600	SEM	p-value
**Feed Intake, g/ bird/ day**						
23-26 wk	114.0	114.1	114.4	116.3	0.84	0.745
27-30 wk	110.3^b^	113.2^ab^	116.5^a^	117.8^a^	0.77	<0.001
31-34 wk	112.3^b^	112.7^b^	117.8^a^	118.9^a^	0.72	<0.001
35-38 wk	113.4^b^	106.0^c^	115.6^ab^	117.3^a^	0.93	<0.001
39-44 wk	111.6^a^	98.6^b^	113.1^a^	113.4^a^	1.34	<0.001
Overall	112.3^bc^	108.9^c^	115.5^ab^	116.8^a^	0.70	<0.001
**Feed Conversion Ratio (g feed/g egg mass)**						
23-26 wk	2.17	2.09	2.16	2.15	0.02	0.325
27-30 wk	1.91	1.96	1.98	1.97	0.01	0.057
31-34 wk	1.87^b^	1.91^ab^	1.96^a^	1.95^a^	0.01	0.012
35-38 wk	1.87^b^	1.95^a^	1.92^ab^	1.93^ab^	0.01	0.013
39-44 wk	1.90^b^	2.28^a^	1.95^b^	1.95^b^	0.04	<0.001
Overall	1.94^b^	2.05^a^	2.00^ab^	2.00^ab^	0.01	0.002

a-c Means with no common letters in the same row differ significantly (p < 0.05).

^1^Data represent mean values of 9 replicates per treatment.

^2^PC, positive control (nutritionally adequate diet based on breeder nutrient recommendations); NC, negative control (the PC diet with reduced metabolizable energy 2630 vs. 2730 kcal/kg, and available phosphorus levels 0.12 vs 0.38%); NC300 and NC600, the NC diets supplemented with hybrid novel consensus bacterial 6-phytase (Natuphos E^®^ 10000) at 300 and 600 FTU/kg, respectively.

The dietary treatments had no significant effect on FCR (p > 0.05) during the first two periods (23–26 and 27–30 wk). During the whole trial (23–44 wk), especially in the last two periods (35–38 wk and 39–44 wk), the nutritional reductions in NC group adversely affected FCR compared to hens fed the PC diet (p < 0.05). The supplementation of phytase at both levels alleviated this negative effect, resulting in FCR comparable to that of the PC group (p < 0.05). At the end of trial, hens fed NC diets had significantly lower body weight (p < 0.001) and higher mortality (p < 0.05) compared to those fed PC, NC300, and NC600 diets (Table 4).

**Table 4:** The effects of phytase supplementation on body weight and cumulative mortality in hens on low phosphorus and low-energy diets^1^.

	Body Weight, g		Mortality, %
**Treatment** ^2^	**23 wk**	**44 wk**	**23-44 wk**
**PC**	1827.4	1938.4^a^	3.70^b^
**NC**	1820.6	1749.1^b^	9.26^a^
**NC300**	1823.8	1924.2^a^	1.85^b^
**NC600**	1851.8	1935.3^a^	2.78^b^
**SEM**	11.61	16.35	–
**p-value**	0.787	<0.001	0.036

a, b Means with no common letters in the same row differ significantly (p < 0.05).

^1^Data represent mean values of 9 replicates per treatment.

^2^PC, positive control (nutritionally adequate diet based on breeder nutrient recommendations); NC, negative control (the PC diet with reduced metabolizable energy 2630 vs. 2730 kcal/kg, and available phosphorus levels 0.12 vs 0.38%); NC300 and NC600, the NC diets supplemented with hybrid novel consensus bacterial 6-phytase (Natuphos E^®^ 10000) at 300 and 600 FTU/kg, respectively.

### Egg quality characteristics

The impact of phytase supplementation on the incidence of cracked, dirty and shell-less eggs is presented in Table 5. From wk 31 onwards, the nutritional reductions in NC group resulted in an increased number of cracked and shell-less eggs compared to the PC group (p < 0.05). Phytase supplementation at both levels lowered the number of cracked and shell-less eggs compared to NC group (p < 0.05).

**Table 5:** The effects of phytase supplementation on incidences of cracked, dirty and shell-less eggs in hens on low phosphorus and low-energy diets^1^.

	Treatment Groups^2^				Statistics	
	PC	NC	NC300	NC600	SEM	p-value
**Cracked, %**						
23-26 wk	0.79	0.89	0.75	0.74	0.12	0.942
27-30 wk	0.52	0.66	0.38	0.60	0.11	0.567
31-34 wk	0.42^b^	1.02^a^	0.60^ab^	0.60^ab^	0.13	0.023
35-38 wk	0.54^b^	2.02^a^	0.72^b^	0.90^b^	0.19	<0.001
39-44 wk	0.49^b^	3.65^a^	0.38^b^	0.53^b^	0.38	<0.001
Overall	0.55^c^	1.65^a^	0.56^c^	0.67^b^	0.12	<0.001
**Dirty, %**						
23-26 wk	0.20	0.26	0.29	0.16	0.06	0.711
27-30 wk	0.45	0.56	0.26	0.38	0.07	0.359
31-34 wk	0.48	0.44	0.35	0.29	0.07	0.663
35-38 wk	0.60^a^	0.54^a^	0.44^ab^	0.12^b^	0.08	0.009
39-44 wk	0.35	0.72	0.33	0.62	0.07	0.082
Overall	0.41	0.50	0.33	0.33	0.04	0.115
**Shell-less, %**						
23-26 wk	0.97	0.92	0.81	1.12	0.14	0.530
27-30 wk	0.39	0.52	0.32	0.41	0.08	0.729
31-34 wk	0.16^b^	0.61^a^	0.16^b^	0.22^b^	0.09	0.013
35-38 wk	0.16^b^	0.72^a^	0.09^b^	0.12^b^	0.08	<0.001
39-44 wk	0.12^b^	0.81^a^	0.10^bc^	0.10^bc^	0.07	<0.001
Overall	0.34^b^	0.72^a^	0.28^b^	0.37^b^	0.06	<0.001

a-c Means with no common letters in the same row differ significantly (p < 0.05).

^1^Data represent mean values of 9 replicates per treatment.

^2^PC, positive control (nutritionally adequate diet based on breeder nutrient recommendations); NC, negative control (the PC diet with reduced metabolizable energy 2630 vs. 2730 kcal/kg, and available phosphorus levels 0.12 vs 0.38%); NC300 and NC600, the NC diets supplemented with hybrid novel consensus bacterial 6-phytase (Natuphos E^®^ 10000) at 300 and 600 FTU/kg, respectively.

Egg shape index and eggshell thickness (Table 6) were unaffected by dietary treatments (p > 0.05). During the 31–34 and 39–44 wk periods, eggshell breaking strength was significantly lower in the NC group compared to PC (Table 6), and phytase supplementation restored the shell strength to levels comparable to the PC group (p < 0.05). There were no noticeable differences among treatments in terms of yolk index (p > 0.05) (Table 7). No statistical differences were observed among treatments for albumin index and Haugh Units, except during the last period (39–44 wk), where both parameters were higher in the NC group compared to the PC, NC300, and NC600 groups (p < 0.05) (Table 7). During the initial period (23–27 wk), yolk color score was higher for the PC group compared to NC, NC300 and NC600 groups (p < 0.05). However, for the remaining periods, yolk color scores were comparable across all treatments (p > 0.05) (Table 7).

**Table 6:** The effects of phytase supplementation on egg shape index, breaking strength, shell thickness, and Haugh unit in hens on low phosphorus and low-energy diets^1^.

	Treatment Groups^2^				Statistics	
	PC	NC	NC300	NC600	SEM	p-value
**Shape Index, %**						
23-26 wk	81.42	80.39	80.81	80.03	0.25	0.224
27-30 wk	80.31	80.78	80.33	79.64	0.26	0.502
31-34 wk	79.22	79.89	79.39	79.53	0.27	0.852
35-38 wk	78.81	79.83	78.94	79.19	0.26	0.522
39-44 wk	78.92	78.92	79.86	78.19	0.25	0.128
**Breaking Strength, kg/cm** ^**2**^						
23-26 wk	4.23	4.16	4.22	4.24	0.10	0.990
27-30 wk	4.54	4.06	4.32	4.33	0.11	0.514
31-34 wk	4.51^a^	3.76^b^	4.48^ab^	4.28^ab^	0.10	0.036
35-38 wk	3.92	3.52	4.28	4.19	0.12	0.114
39-44 wk	4.43^a^	3.37^b^	3.97^ab^	4.21^a^	0.11	0.005
**Shell Thickness, µm**						
23-26 wk	371.6	381.4	373.9	374.5	2.64	0.595
27-30 wk	373.1	360.8	373.4	369.2	2.56	0.275
31-34 wk	358.5	347.2	354.9	361.2	2.29	0.153
35-38 wk	386.1	376.8	382.1	385.9	2.79	0.624
39-44 wk	371.3	352.6	365.8	364.4	3.80	0.365

a, b Means with no common letters in the same row differ significantly (p < 0.05).

^1^Data represent mean values of 18 replicates per treatment.

^2^PC, positive control (nutritionally adequate diet based on breeder nutrient recommendations); NC, negative control (the PC diet with reduced metabolizable energy 2630 vs. 2730 kcal/kg, and available phosphorus levels 0.12 vs 0.38%); NC300 and NC600, the NC diets supplemented with hybrid novel consensus bacterial 6-phytase (Natuphos E^®^ 10000) at 300 and 600 FTU/kg, respectively.

**Table 7:** The effects of phytase supplementation on yolk index, albumin index, and yolk color score in hens on low phosphorus and low-energy diets^1^.

	Treatment Groups^2^				Statistics	
	PC	NC	NC300	NC600	SEM	p-value
**Yolk Index, %**						
23-26 wk	48.84	47.44	47.91	47.22	0.29	0.213
27-30 wk	45.50	44.31	44.65	45.07	0.27	0.438
31-34 wk	43.84	43.01	43.88	43.61	0.27	0.666
35-38 wk	41.87	41.54	41.70	42.38	0.30	0.780
39-44 wk	43.25	44.37	43.67	43.66	0.24	0.428
**Albumin Index, %**						
23-26 wk	10.99	10.41	10.6	10.58	0.19	0.756
27-30 wk	9.29	9.76	9.37	9.40	0.22	0.888
31-34 wk	8.55	9.12	8.84	8.74	0.16	0.664
35-38 wk	8.62	9.07	8.71	9.53	0.24	0.531
39-44 wk	7.98^b^	9.94^a^	8.12^b^	8.43^b^	0.21	0.002
**Haugh Unit**						
23-26 wk	90.89	88.83	89.61	89.28	0.60	0.660
27-30 wk	84.49	85.38	84.69	84.58	0.89	0.986
31-34 wk	82.41	83.89	82.83	82.90	0.59	0.845
35-38 wk	81.26	84.42	82.75	86.20	0.99	0.334
39-44 wk	80.25^b^	86.75^a^	80.52^b^	81.86^b^	0.85	0.021
**Yolk Color Score**						
23-26 wk	12.53^a^	11.72^b^	11.92^b^	11.61^b^	0.08	<0.001
27-30 wk	10.89	11.25	11.31	11.42	0.09	0.209
31-34 wk	11.08	11.00	11.00	11.17	0.07	0.782
35-38 wk	10.06	10.19	10.36	10.14	0.09	0.657
39-44 wk	11.06	11.06	11.14	10.92	0.07	0.694

a, b Means with no common letters in the same row differ significantly (p < 0.05).

^1^Data represent mean values of 18 replicates per treatment.

^2^PC, positive control (nutritionally adequate diet based on breeder nutrient recommendations); NC, negative control (the PC diet with reduced metabolizable energy 2630 vs. 2730 kcal/kg, and available phosphorus levels 0.12 vs 0.38%); NC300 and NC600, the NC diets supplemented with hybrid novel consensus bacterial 6-phytase (Natuphos E^®^ 10000) at 300 and 600 FTU/kg, respectively.

### Tibia quality characteristics

The impact of phytase supplementation on tibia characteristics is presented in Table 8. The tibia ash content, stiffness and bone breaking strength were adversely affected by nutritional reductions in the NC diet compared to the PC group (p < 0.05). Phytase addition at both levels restored the tibia ash content, stiffness and bone breaking strength to levels comparable to the PC group (p < 0.05).

**Table 8:** The effects of phytase supplementation on some tibia characteristics in hens on low phosphorus and low-energy diets^1^.

	Treatment Groups^2^				Statistics	
Item^3^	PC	NC	NC300	NC600	SEM	p-value
**Ash, %**	53.38^a^	49.36^b^	54.49^a^	54.38^a^	0.39	<0.001
**Stiffness, N/mm**	250.6^a^	193.5^b^	259.4^a^	248.1^a^	7.68	0.005
**Breaking strength, N**	130.0^ab^	115.6^b^	143.4^a^	138.4^a^	3.75	0.040
**RANKL** ^**4**^	18.41^b^	22.93^a^	18.41^b^	19.63^b^	0.38	<0.001
**OPG** ^**4**^	25.48^a^	18.89^c^	23.07^b^	21.96^b^	0.43	<0.001
**RANKL/OPG**	0.72^c^	1.21^a^	0.80^c^	0.89^b^	0.03	<0.001

a-c Means with no common letters in the same row differ significantly (p < 0.05).

^1^Data represent mean values of 9 replicates per treatment (18 replicates/treatment for Ash, %).

^2^PC, positive control (nutritionally adequate diet based on breeder nutrient recommendations); NC, negative control (the PC diet with reduced metabolizable energy 2630 vs. 2730 kcal/kg, and available phosphorus levels 0.12 vs 0.38%); NC300 and NC600, the NC diets supplemented with hybrid novel consensus bacterial 6-phytase (Natuphos E^®^ 10000) at 300 and 600 FTU/kg, respectively.

^3^RANKL, Receptor Activator of Nuclear Factor Kappa-B Ligand; OPG, Osteoprotegerin.

^4^Number of immunopositive cells.

**Fig 1:**
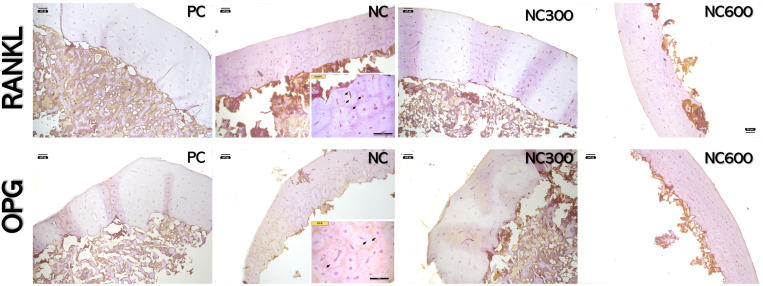
Immunohistochemical staining of tibia samples showing the effects of phytase supplementation on OPG and RANKL expressions in hens. The arrows indicate immunopositive cells in tibia. Scale bar: 50 µm.

Immunohistochemical analysis (Table 8 and Fig 1) of the tibia revealed a higher expression of RANKL protein in hens fed the NC diet than in the PC and phytase-supplemented groups (p < 0.001). Conversely, the OPG expression was highest in the PC group than NC, and the addition of phytase to NC diets significantly increased the OPG positive cells compared to the non-supplemented NC group (p < 0.001). The RANKL/OPG ratio was significanly highest in the NC group, followed by NC600, NC300 and PC (p < 0.001).

## Discussion

Microbial phytases are widely recognized for their role in enhancing phytate degradation, thereby increasing the availability of plant-derived P in poultry diets, while also improving the digestibility of other nutrients. With the ongoing development of novel exogenous phytases, it is important to continuously assess their efficacy when added to poultry diets. This study evaluated the potential of a hybrid novel consensus bacterial 6-phytase variant to completely replace dietary inorganic P in low-energy laying hen diets during their first production cycle (23–44 weeks of age) and assessed its impact on hen performance, egg quality, and bone health.

The present study demonstrated that hens fed a diet lacking supplementation of inorganic P and exogenous phytase (NC group: 0.12% nPP and 2630 kcal/kg ME) exhibited significantly impaired performance (HHEP, HDEP, egg weight, egg mass, FI, FCR, body weight and livability) compared to those on a nutritionally adequate diet (PC group: 0.38% nPP and 2730 kcal/kg ME). However, phytase supplementation to NC diets at both tested levels (300 and 600 FTU/kg) effectively restored these production parameters, highlighting its role in improving P utilization and overall hen performance. These findings align with previous studies showing that inadequate dietary nPP levels (0.10–0.12%) impair P and other nutrient utilization, leading to reduced egg production, poor skeletal health, and lower metabolic efficiency [17,23,39–45]. This is attributed to the inability of hens to hydrolyze phytate-bound P efficiently, which limits P availability for metabolic functions. Boling et al. [40] found that while White Leghorn hens (20–70 wk) maintained egg production at 0.15% nPP without phytase, their body weight and tibia were negatively affected, showing the long-term skeletal implications of P deficiency, even when egg production is sustained. Similarly, research on Hy-Line brown and Lohmann hens [16,46] indicated that reductions in nPP levels to 0.14–0.15% without phytase led to decreased egg production, likely due to inadequate P for eggshell formation and cellular metabolism. A meta-analysis by Rodehutscord et al. [20] suggested that hens may require no more than 0.22% nPP in diets without phytase. Assuming a daily FI of 110–120 g/hen, this equates to a daily nPP intake of 242–264 mg/hen/day. Thus, variability in the literature regarding adequate nPP levels in laying hen diets without phytase supplementation can be attributed to differences in nutrient composition and digestibility, as well as factors such as feeding duration and bird age.

In this study, phytase supplementation (300 and 600 FTU/kg) enabled the complete removal of inorganic P without compromising performance. It was consistent with findings from Panda et al. [19] and Punna and Roland [43], who demonstrated that adding 300–500 FTU/kg phytase alleviated P deficiency symptoms in laying hens fed diets with 0.10–0.12% nPP. Punna and Roland [43] further noted that phytase supplementation had minimal effects when available P levels exceeded 0.20%, reinforcing the notion that phytase effectiveness is highly dependent on baseline P levels. Additionally, research involving laying hens (0.12–0.14% nPP) has shown potential of phytase inclusion (250–2000 FTU/kg) to compensate for multiple nutrient reductions, i.e., ME (50–75 kcal/kg), calcium (~0.2pp), and protein (0.2–0.8 pp) without adverse effects [15–17]. Moura et al. [17] observed better egg production at higher phytase inclusion levels (600–900 FTU/kg), attributed to low mortality rates. Collectively, these findings emphasize the need to optimize phytase inclusion levels and make dietary adjustments beyond P alone to maximize phytase efficiency and reduce the environmental footprint of poultry production.

Ahmadi et al. [22], in a meta-analysis, suggested that optimal dietary nPP levels for laying hens could be lowered to 0.15% with the inclusion of 300 FTU/kg of phytase. Our findings corroborate this perspective, demonstrating that nPP levels can be further reduced by 0.03 pp, alongside a 100 kcal/kg reduction in ME content, in phytase supplemented diets (300 and 600 FTU/kg) for Lohmann Brown hens during 23–44 wks. Further research is essential to refine our understanding of the nutritional matrices associated with bacterial 6-phytase supplementation to ensure the maintenance of adequate nutrient intake and optimize performance in laying hens.

Egg weight is a key criterion in egg grading, with studies showing a positive correlation between egg weight and the body weight of the hen [47]. In this experiment, the negative effects of nPP deficiency and reduced ME on egg production became apparent after eight weeks, consistent with early findings [40,41]. Regarding egg weight, apart from an initial pronounced response to nutrient variation, all the treatment groups exhibited consistent trends until the 38^th^ week (16 wk into trial). However, between weeks 39–44, the homeostasis mechanisms in hens fed NC diet appeared to be disrupted, leading to a significant decline in egg weight compared to the PC. This reduction coincided with the lowest FI (98.60 g/bird/day), and the lowest P intake (118.32 mg/bird/day) recorded for NC birds. Trends in egg mass mirrored those observed in egg production and egg weight across different production periods. Previous studies [15,17,24,42,43,45,48] have shown that feeding laying hens diets low in nPP (0.10–0.14%) decreases egg weight and/or egg mass compared to nutritionally adequate diets, however, these negative effects are alleviated by phytase supplementation. The current study supports these findings, showing that phytase supplementation significantly improved both egg weight and egg mass in NC diets, making them comparable to the PC group. Notably, a dose of 600 FTU/kg yielded better results for egg weight than 300 FTU/kg. This improvement may be attributed to phytase-induced nutritional modifications affecting egg composition.

The current findings align with previous research demonstrating that nutrient down-specification, particularly low nPP levels, negatively impacts FI in laying hens compared to nutritionally adequate diets, and the inclusion of phytase in these diets effectively mitigates such decline [16,42,43,45,48,49]. Consistent with Pirzado et al. [15], our study observed a heightened FI response at higher phytase dosage. Based on the expected nPP contribution from phytase (162 and 210 mg/hen/day for 300 and 600 FTU/kg, respectively) and the observed FI during weeks 23–44, the estimated daily net nPP availability per hen was approximately 427 mg (PC), 131 mg (NC), 300 mg (NC300) and 350 mg (NC600). These values with phytase supplementation exceeded the net nPP availability reported by Rama Rao et al. [24] which ranged from 258–264 mg/hen/day for diets containing 0.12% nPP + 600 FTU/kg phytase. These results affirm the view that a dietary nPP reduction of 0.10% to 0.20% could be achieved with phytase supplementation [50]. Notably, the PC group had an estimated oversupply of nPP of approximately 42.0% compared to the NC300, yet performance outcomes were comparable. Similarly, the total energy intake per hen per day was estimated at 307 kcal (PC), 286 kcal (NC), 311 kcal (NC300), and 317 kcal (NC600), further illustrating the potential of phytase to offset nutrient deficits.

Our findings also support previous studies indicating that while phytase supplementation enhances feed consumption by increasing nPP and energy availability, thereby improving laying performance, its effect on FCR remains minimal [16,49]. In contrast, Pirzado et al. [15] reported that phytase improved FCR, even surpassing the control group. Overall, it is important to emphasize that the effects of phytase on FI and FCR may vary depending on several factors, including diet composition, enzyme dosage, bird age, body weight, duration of nutrient restriction, and environmental conditions.

The literature regarding the effects of nPP content or phytase supplementation on various egg quality parameters is inconsistent. A higher incidence of broken or cracked eggs was observed when nPP levels in laying hen diets were reduced to 0.15% or lower without phytase supplementation [51]. Similarly, in this study, decreasing dietary nPP and energy levels (NC) resulted in more cracked and shell-less eggs, along with reduced shell breaking strength. These adverse effects were mitigated by phytase supplementation, which aligns with findings from other studies [23,24,45,51]. However, research also reported no effect of nPP content or phytase supplementation on cracked egg rates, eggshell thickness, or shell breaking strength [15–17,49]. Some studies noted improvements in eggshell thickness with phytase supplementation [45,46]. Previous studies have linked nPP content and phytase supplementation to improve yolk color [52], although low nPP levels have also been associated with increased yolk color scores [15,17]. However, we did not observe any such changes. Discrepancies in yolk color findings may stem from variations in feed ingredient composition across studies. In this study, the NC group exhibited increased albumin height and Haugh units compared to PC and phytase supplemented groups. This may be explained by lighter eggs in the NC group, where changes in albumin to yolk ratio could have contributed to higher albumin height and Haugh unit values. However, some researchers did not find any effect of nPP levels on albumin height or Haugh unit [15,49]. Further research is needed to clarify the impact of nPP content and phytase supplementation on the nutrient composition of egg yolk and albumin, as well as their overall influence on egg quality traits.

Bone ash is an essential measure for assessing bone quality, particularly during mineral scarcity. The significant decrease in tibia ash percentage observed in this study suggests that hens on NC diets (0.12% nPP) without phytase mobilized minerals from their bone to compensate for P deficiency. Phytase supplementation at both dosage levels not only prevented this bone loss but also enhanced tibia mechanical properties, including resistance to deformation and maximum load bearing capacity, matching the PC group. These findings align with earlier research where phytase improved bone ash content and strength when supplemented to hen diets with low nPP (0.10–0.15%) concentrations [16,24,48,53]. To further assess the impact of reduced nPP levels and phytase supplementation on bone health, this study examined RANKL and OPG, key regulators of bone formation and resorption. A significant elevation in the RANKL/OPG ratio was observed in the NC group, indicating increased osteoclast activity and accelerated bone resorption. This imbalance, characterized by increased osteoclastogenesis, is a hallmark of bone loss under phosphorus-deficient conditions. Phytase supplementation, by increasing dietary P availability, corrected this imbalance and promoted a more anabolic bone environment. Teng et al. [54] investigated the effects of P on osteoblasts and osteoclasts balance, observing reduced OPG and increased RANKL protein expression in the tibia of hens fed low P diets. Other studies have similarly shown that P-deficient diets promote osteoclasts formation and reduce bone mineralization and strength in laying hens [55,56]. Overall, it may be suggested that supplementing phytase (300–600 FTU/kg) to P-deficient diets (0.12% nPP) could prevent bone loss by modulating specific markers involved in bone metabolism and remodeling.

## Conclusion

In conclusion, this study demonstrated that the hybrid bacterial 6-phytase variant effectively mitigated the negative impacts of low nPP (0.12% nPP) and reduced ME (−100 kcal/kg) in laying hen diets (NC group), offering an alternative to inorganic P supplementation. The results showed that phytase supplementation at both levels (300 and 600 FTU/kg) restored performance (egg production, egg weight, FI, FCR, body weight, livability), egg quality (incidences of cracked and shell-less eggs, shell breaking strength, albumin index, Haugh unit), bone health (tibia ash, stiffness, breaking strength, and bone metabolic markers, including the RANKL/OPG ratio) to levels comparable to the PC group. Additionally, the 600 FTU/kg supplementation provided slightly better results in terms of egg weight and eggshell strength than the 300 FTU/kg level. Nonetheless, further research is recommended to determine the optimal levels of hybrid bacterial 6-phytase in P-deficient laying hen diets while assessing other nutrient matrices, such as calcium levels, along with variations in energy and amino acid densities to optimize dietary formulations.
